# The Effects of Purple Corn Pigment on Growth Performance, Blood Biochemical Indices, Meat Quality, Muscle Amino Acids, and Fatty Acids of Growing Chickens

**DOI:** 10.3390/foods11131870

**Published:** 2022-06-24

**Authors:** Qingyuan Luo, Jiaxuan Li, Hui Li, Di Zhou, Xu Wang, Yayuan Tian, Jixiao Qin, Xingzhou Tian, Qi Lu

**Affiliations:** 1 Key Laboratory of Animal Genetics, Breeding and Reproduction in the Plateau Mountainous Region, Ministry of Education, College of Animal Science, Guizhou University, Guiyang 550025, China; natty579@163.com (Q.L.); jiaxuanli8899@163.com (J.L.); ellenlihui@sina.cn (H.L.); wangxu10216610@163.com (X.W.); yytian17385827096@163.com (Y.T.); zrz2942037436@163.com (J.Q.); xztian3@gzu.edu.cn (X.T.); 2 Testing Center for Livestock and Poultry Germplasm, Guizhou Agricultural and Rural Affairs Office, Guiyang 550018, China; dizhougz@163.com

**Keywords:** purple corn anthocyanin, growth performance, antioxidant activity, meat quality, amino acid, fatty acid

## Abstract

**Simple Summary:**

Anthocyanin is a large type of polyphenol compound that is a strong natural antioxidant and widely exists in natural plant and food fields. Specifically, anthocyanins can relieve oxidative stress by scavenging oxygen free radicals to improve meat quality and extend the shelf life of meat products. Chicken meat contains high-quality protein and other substances important in the human diet and has become quite popular among consumers. Therefore, anthocyanin is a natural pigment that has been extremely valuable. We hypothesized that anthocyanin-rich purple corn pigment can improve growth performance, enhance antioxidant activity, and improve meat quality, muscle amino acid, and fatty acid profiles in chickens. The results of the current study indicate that the consumption of anthocyanin-rich purple corn pigment by growing chickens has the potential to increase growth performance, enhance antioxidant and immune capacities, heighten meat quality, and improve essential amino acids, umami amino acids, and unsaturated fatty acids in muscle.

**Abstract:**

This study investigated the effects of dietary supplementation with different levels of purple corn pigment (PCP) on the growth performance, blood biochemical indices, meat quality, muscle amino acids, and fatty acids of growing chickens. A total of 288 (8 weeks of age) growing Chishui black-bone chickens (body weight, 940 ± 80 g; mean ± standard deviation) were randomly divided into 4 groups using a completely randomized design. The four diet groups were as follows: (1) control, basal diet; (2) treatment 1, treatment 2, and treatment 3, which were basal diet with 80, 160, and 240 mg/kg PCP, respectively. The results showed that compared with the control group, the feeding of anthocyanins significantly (*p* < 0.05) increased the average daily feed intake and average daily gain in chickens. Moreover, chickens receiving 80 mg/kg PCP significantly increased (*p* < 0.05) plasma total antioxidant capacity, superoxide dismutase, glutathione peroxidase, catalase, high-density lipoprotein cholesterol, and albumin concentrations relative to the control group. For meat quality, dietary supplementation with PCP significantly (*p* < 0.05) reduced the drip loss and water loss rate in breast muscle. Additionally, chickens receiving PCP tended to increase (*p* < 0.05) the levels of most individual amino acids, essential amino acids, and umami amino acids in the muscle. Specifically, the addition of 80 mg/kg PCP significantly improved (*p* < 0.05) total polyunsaturated fatty acids in chicken muscle. Accordingly, the consumption of anthocyanin-rich PCP by the growing chickens had the potential to increase the growth performance, enhance antioxidant and immune capacities, increase meat quality, and improve essential and umami amino acids as well as unsaturated fatty acids in the muscle.

## 1. Introduction

Poultry products are ideal for human nutrition and are indispensable in people’s lives; they have good potential to supply animal protein because of their rapid growth and high productivity [1]. Oxidative stress is an imbalance between the production of cell-damaging free radicals and the body’s ability to neutralize them; various factors lead to oxidative stress in poultry farms, such as the stocking density, diet composition, and feeding system [2]. Thus, the content of free radicals could increase when the body is under oxidative stress, affecting animal health and leading to a decline in the quality of meat products and the efficiency of farming [3]. Notably, a previous study showed that plant extracts could be used as antibiotic alternatives in poultry diets [4]. Flavonoids are natural antioxidant extracts that can relieve oxidative stress by scavenging oxygen free radicals to improve meat quality and extend the shelf life of meat products [5,6]. Moreover, one study showed that feeding flavonoid-rich thymol and carvacrol can enhance the growth performance, antioxidant enzyme activities, digestive enzyme activities, and immune response of Ross-308 male broiler chickens [7].

Anthocyanins are a source of water-soluble flavonoid pigments in plants which have antioxidant, antibacterial, anti-inflammatory, and anticancer effects [8]. Kšonžeková et al. [9] showed that anthocyanins could maintain the balance of oxidation state in the production process of the body, improving the health of livestock and poultry. Specifically, there are abundant anthocyanin compounds in purple corn, showing a wide range of biological activities in terms of antioxidation, scavenging free radicals, antitumour activities, cardiovascular protection, and improving microcirculation [10,11]. Moreover, purple corn had a higher total anthocyanin content and stronger 2,2-diphenyl-1-picrylhydrazine (DPPH) scavenging activity and antioxidant activity in vitro [12]. Amer et al. [13] found that the inclusion of anthocyanin-rich roselle, *Hibiscus sabdariffa* L. extract, could improve chicken antioxidant potential by increasing serum total antioxidant capacity (TAC) and superoxide dismutase (SOD) concentrations and by decreasing serum malondialdehyde (MDA) level. Additionally, Hajrawati et al. [14] demonstrated that anthocyanin-rich purple sweet potato flour could increase the cooking loss, anthocyanin content, redness, and scavenging activity of chicken meatballs.

Purple corn pigment (PCP) is an important source for the development of natural antioxidants due to it containing an abundance of anthocyanins, which is very crucial for animal health because it had good pigmentation and physiologic function in antioxidation [11,15]. To our knowledge, there are limited studies on the effect of PCP as a feed additive on meat quality, meat amino acid, and fatty acid profiles in chickens. Chishui black-bone chicken is a unique local characteristic poultry in Chishui City, Guizhou Province, China, which has very high research value because of its delicious taste, rich nutrition, and its growing popularity with consumers [16]. We hypothesized that anthocyanin-rich PCP can improve growth performance, enhance antioxidant activity, and improve meat quality, muscle amino acid, and fatty acid profiles in chickens. Accordingly, the aim of the present study was to observe the effect of anthocyanin-rich PCP on the growth performance, blood biochemical indices, meat quality, muscle amino acid, and fatty acid profiles in growing Chishui black-bone chickens.

## 2. Materials and Methods

### 2.1. Experimental Design and Management

Farahat et al. [17] found that grape seed anthocyanin extract can be used as an effective natural antioxidant and the optimum dosage was 125 to 250 mg/kg in broiler chicken diet. As a result, this study was conducted in a commercial Chishui black-bone chicken farm (28.590337 N, 105.697472 E, Chishui, China). Using a completely randomized design, a total of 288 (8 weeks of age) growing Chishui black-bone chickens with similar body weight (BW, 940 ± 80 g; mean ± standard deviation) were randomly divided into 4 groups with 6 replicates, with each replicate having 12 chickens. The control group (CON) was fed a basal diet, and treatment 1 (LA), treatment 2 (MA), and treatment 3 (HA) were fed a basal diet with 80, 160, and 240 mg/kg anthocyanin-rich PCP, respectively. The PCP was purchased from the Nanjing Herd Source Biotechnology Co., Ltd. (Nanjing, China), its anthocyanin composition was detected by a high-performance liquid chromatography tandem mass spectrometry machine, and the total anthocyanin content was 2.62 mg/g according to our previous studies [15,18]. The feeding trial period lasted for 75 d, which comprised a preparation period of 15 d, and a formal experimental period was 60 d. All the experimental chickens were kept in clean individual chicken coops, and the coops were kept in light for 16 h per day with constant temperature (23 °C) and humidity (60%). All experimental chickens were fed at 05:00 and 17:00 with *ad libitum* intake, and water was freely provided. The basal diet was based on the Feeding Standard of Chicken (NY/T 33-2004) [19], and the composition and nutrient level of the basal diet are presented in Table 1.

### 2.2. Chemical Composition

The chemical composition of feed for dry matter, crude protein, calcium, and phosphorus was determined by the method of the Association of Official Analytical Chemists [20]. Additionally, the amino acids were detected in the feed according to the Chinese Standard GB 5009. 124-2016 [21].

### 2.3. Growth Performance

The consumption of feed was recorded every day to calculate the average daily feed intake (ADFI). In addition, BW was weighed on the first day and the last day to calculate body weight change (BWC) and average daily gain (ADG). The feed/gain (F/G) was calculated at the end of the experiment. The growth performance parameters were calculated according to Omar [22] as follows: ADFI (g/d) = cumulative feed intake/(number of birds × number of days); BWC (g) = initial weight (g)−final weight (g); ADG (g/d) = BWC (g)/number of days (d); and F/G = feed intake (g)/weight gain (g).

### 2.4. Blood Biochemical Indices

On the 20th, 40th, and 60th days of the formal experimental period, twelve chickens per group were selected randomly, and 5 mL of blood was collected from the underwing vein using heparin sodium anticoagulation tubes. The blood samples were centrifuged at 4000× *g* for 15 min (KJH80-2, Jiangsu Kang Jianhua Medical Supplies Co., Ltd., Taizhou, China), and the above plasma was transferred to a 1.5-mL centrifuge tube and stored at −80 °C until further analysis. The antioxidant parameters TAC (code No. A015-1-2), SOD (code No. A001-3-2), glutathione peroxidase (GSH-Px; code No. A005-1-2), catalase (CAT; code No. A007-1-1), and MDA (code No. A003-1-2); the lipid metabolism parameters total cholesterol (T-CHO; code No. A111-1-1), triglyceride (TG; code No. A110-1-1), low-density lipoprotein cholesterol (LDL-C; code No. A113-1-1), and high-density lipoprotein cholesterol (HDL-C; code No. A112-1-1); the immune parameters total protein (TP; code No. A045-4-2) and albumin (Alb; code No. A028-2-2) were determined using commercial kits (Nanjing Jiancheng Bioengineering Co., Ltd., Nanjing, China). All procedures were strictly completed in accordance with reagent instructions. Data were acquired by a microplate reader using MPM 6.1 software (Synergy H4. BioTek, Winooski, VT, USA).

### 2.5. Meat Quality

At the end of the feeding experimental period, six chickens were slaughtered in each group, and breast muscle was separated, trimmed, and weighed from each carcass for meat quality examination. The pH value was detected at 45 min and 24 h using a pH-star equipment (Eckelsheim, Germany) after calibration using standard buffer solutions with pH 4.0 and 7.0. The meat color (opto) was detected at 45 min and 24 h after slaughter by an opto-star machine (Eckelsheim, Germany). Before measurement, the opto-star machine was calibrated according to the specific calibration module and the system was defined as the L-value (L*). For drip loss, the fresh meat sample was weighed (recorded as W1), suspended and sealed, placed in a 4 °C refrigerator for 24 h, and weighed again (recorded as W2) to calculate the drip loss, and drip loss (%) = [(W1−W2)/W1] × 100. For the water loss rate, the fresh meat sample was weighed (recorded as W3), covered with filter papers on both sides, pressed with 30 kg of gravity for 30 min (Tenovo Meat-1, Beijing, China), and then weighed again (recorded as W4) to calculate the water loss rate, and water loss rate (%) = [(W3−W4)/W3] × 100. For shear force (N), approximately 2 g of the meat sample (2 cm × 1 cm × 1 cm) was weighed and was sheared perpendicular to the grain of the muscle fiber with a load cell of 15 kg and a 200 mm/min crosshead speed using a tenderness meter (Xielikeji Co., Ltd., Harbin, China). Three measurements were determined of each sample for the above five parameters.

### 2.6. Amino Acids and Fatty Acids

After slaughter, breast muscle was immediately separated and stored at −80 °C, and then all samples were lyophilized (LYOQUEST-85 PLUS, Telstar Industry, Spain) for the determination of amino acids and fatty acids.

The individual amino acids in the breast muscle were determined as per the method of the National Standard for Determination of Fatty Acids in Food (GB 5009.168–2016) [21]. Briefly, 15 mL of 6 mol/L hydrochloric acid solution and 4 drops of phenol were added to a hydrolysis tube, which was put into the refrigerant for 5 min and then connected to the suction tube of the vacuum pump, evacuated (close to 0 Pa), and filled with nitrogen; this process was repeated 3 times. The sealed hydrolysis tube was placed at 110 °C in a hydrolysis furnace, hydrolyzed for 22 h, and cooled to room temperature. The individual amino acids were detected by an amino acid automatic analyzer (Model: Biochrom 30, Biochrom Ltd., Cambridge, UK) at wavelengths of 440 nm–570 nm. 16 kinds of amino acids were as follows: asparagine (Asp), threonine (Thr), serine (Ser), glutamic acid (Glu), glycine (Gly), alanine (Ala), valine (Val), methionine (Met), isoleucine (Ile), leucine (Leu), tyrosine (Tyr), phenylalanine (Phe), histidine (His), lysine (Lys), arginine (Arg), and proline (Pro). The detection signal was UV 338 nm, fluorescence (EX = 266 nm, EM = 305 nm); mobile phase A was 40 mmol/L sodium dihydrogen phosphate (pH = 7.8); mobile phase B contained acetonitrile, methanol, and water at ratios of 45, 45, and 10, respectively. All experimental procedures were strictly completed in accordance with standard instructions; the difference of measurement results in less than 10% of the arithmetic mean values, and the correlation coefficient of linear regression equation for each individual amino acid was >0.99.

The individual fatty acids of breast muscle were analyzed based on the Chinese Standard GB 5009.168–2016 [23]. Briefly, approximately 2 g of sample was weighed and transferred to a 50-mL tube, and then 20 mL of chloroform and 10 mL of methanol were added, sonicated for 10 min, and shaken for 2 h. A volume of 6 mL of 0.9% sodium chloride aqueous solution was added and shaken for 30 s and kept in a 4 °C water bath for 22 h. The low chloroform layer was collected after centrifugation at 3500× *g* at 4 °C for 10 min and then filtered by filter paper. The filtrate was placed in a dry flask and dried in a vacuum drying oven. A single standard solution of fatty acid methyl ester and a mixed standard solution of fatty acid methyl ester were injected into the gas chromatograph (GC) to characterize the chromatographic peaks. The individual fatty acids were analyzed by a GC machine (Model: Agilent 6890, USA). The GC conditions were as follows: the capillary column was a polydicyanopropylsiloxane strong polar stationary phase (100 m × 0.25 mm × 0.2 μm). The inject temperature was 270 °C; the detector temperature was 280 °C; the temperature program was 100 °C at the beginning and lasted for 13 min, a 10 °C/min rise to 180 °C and lasted for 6 min, a 1 °C/min rise to 200 °C and lasted for 20 min, and a 4 °C/min rise to 230 °C and lasted for 10.5 min. The carrier gas was nitrogen, the split ratio was 100:1, and the injection volume was 1.0 μL. The peak area normalization method was used for this determination. The whole process was done in strict accordance with standard relative request execution to make sure the experimental data are reliable and accurate; under the condition of repeatability, the difference of measurement results in less than 12% of the arithmetic mean values, and GC chromatogram had good separation and peak shape for standard fatty acids.

### 2.7. Statistical Analysis

All data were analyzed by analysis of variance using the general linear model program of SAS 9.4 (SAS Institute, Inc., Cary, NC, USA). The model is as follows: Y*_ij_*= µ + τ*_i_* + ε*_ij_*, where Y*_ij_* is observation *j*, µ is the overall mean, τ*_i_* is the effect of the treatments, and ε*_ij_* is the random error with a mean of 0 and variance σ^2^. A *p* value of less than 0.05 was considered statistically significant.

## 3. Results

### 3.1. Growth Performance

As shown in Table 2, the PCP supplementation significantly (*p* < 0.05) increased the ADFI and ADG compared with the control group. Similarly, the level of BWC in the LA and MA groups was significantly higher (*p* < 0.05) than that in the CON group. The inclusion of 80 mg/kg PCP had significantly (*p* < 0.05) increased the level of final weight relative to the CON and HA groups. However, PCP supplementation had no effect (*p* > 0.05) on initial weight and F/G values among the four groups.

### 3.2. Blood Biochemical Indices

Compared with the control group, supplementation with PCP significantly increased (*p* < 0.05) the levels of plasma TAC, SOD, and GSH-Px (Table 3). In addition, the CAT level in the 80 mg/kg group was significantly higher (*p* < 0.05) than those in the CON and HA groups. However, there was no difference (*p* > 0.05) in plasma MDA, T-CHO, TG, LDL-C, or TP concentration among the four groups. Differently, the level of HDL-C in the LA group was significantly higher (*p* < 0.05) than those in the other three groups. Furthermore, the feeding of anthocyanin-rich PCP significantly increased (*p* < 0.05) the plasma Alb content relative to the CON group.

### 3.3. Meat Quality

There was no significant difference (*p* > 0.05) in muscle pH or meat color at 45 min and 24 h, and shear force value among the four groups (Table 4). Compared to the CON group, the three treatments significantly decreased (*p* < 0.05) drip loss level in the breast muscle. Furthermore, the muscle water loss rate level in MA and HA groups was significantly higher than that of the CON group.

### 3.4. Amino Acid Contents

As shown in Table 5, the feeding of anthocyanin-rich PCP significantly (*p* < 0.05) improved various individual amino acids (including Asp, Thr, Ser, Glu, Gly, Ala, Val, Ile, Leu, Tyr, Phe, Lys, Arg, and Pro) relative to the CON group. The Met content in LA and MA groups was significantly (*p* < 0.05) higher those in the CON and HA groups. Moreover, chickens receiving 160 and 240 mg/kg anthocyanin-rich PCP had significantly (*p* < 0.05) increased muscle His level in comparison with 0 and 80 mg/kg groups. Compared with the control group, the treatments showed significantly higher (*p* < 0.05) essential amino acid (EAA) levels and umami amino acid (UAA) concentrations in the breast muscle.

### 3.5. Fatty Acid Contents

The feeding of anthocyanin-rich PCP significantly decreased (*p* < 0.05) some individual muscle saturated fatty acid (including SFA, C12:0, C14:0, C15:0, C17:0, C22:0) concentrations compared to the control group (Table 6). There was no significant difference (*p* > 0.05) in muscle C18:2n6t and C18:3n6 levels among all the groups. However, chickens receiving anthocyanin-rich PCP had significantly increased (*p* < 0.05) C18:1n9c and monounsaturated fatty acid (MUFA) contents relative to the control group. In addition, total polyunsaturated fatty acids (PUFAs) were greater (*p* < 0.05) in chickens that received LA than those in the other groups.

## 4. Discussion

Many studies have proven that anthocyanin-rich plant extracts have a positive effect on the growth performance of livestock and poultry [24,25]. For example, Chamorro et al. [26] reported that adding anthocyanin-rich grape pomace extract could improve the growth performance of broilers. In the current study, we found that dietary supplementation with anthocyanin-rich PCP could enhance ADFI and ADG in growing Chishui black-bone chickens, perhaps because anthocyanin-rich plant extract could reduce peristalsis of the intestine and the rate of chyme passing through the small intestine, thereby increasing growth performance [27]. Moreover, the antioxidant properties of PCP improved the complex defense mechanism of the poultry body against oxidative damage; thus, more energy was available for growth performance, which might also be a crucial reason for the increased performance of chickens [28,29]. Consistent with our results, Starčević et al. [30] found that the feeding of anthocyanin-rich plant extracts had the ability to increase growth performance in broiler chickens. Csernus et al. [31] reported the same when anthocyanin supplementation could increase ADFI during the entire experimental period in broiler chickens.

Various reactive oxygen species, reactive nitrogen, peroxides, and other free radicals are produced in animals. However, animals are under oxidative stress conditions when these free radical products exceed their antioxidant buffering capacity, thereby negatively affecting animal health [32]. Generally, the higher the activity of antioxidant enzymes is, the stronger the antioxidant capacity of the body [33]. One study found that flavonoids have a strong ability to scavenge free radicals and enhance antioxidant effects [34]. Anthocyanins are a class of flavonoids in plants that can enhance the antioxidant potential of the body. This is because anthocyanins can not only scavenge oxygen free radicals through phenolic hydroxyl groups but can also improve antioxidant enzyme concentrations in the body, thus improving the body’s antioxidant properties [12]. Specifically, anthocyanin can upregulate antioxidant gene expression levels and downregulate the expression of inflammatory genes in the animal body [35]. Hence, our study found that the inclusion of anthocyanin-rich PCP could improve plasma antioxidant and immune activities, such as TAC, SOD, GSH-Px, CAT, and Alb, in growing chickens, perhaps because anthocyanin-rich plant extracts showed higher levels of DPPH scavenging activity in vitro and reduced the levels of free radicals in the body. Our observations were in agreement with Chamorro et al. [36], who found that adding anthocyanin-rich grape seed extract could enhance the antioxidant properties and delay lipid oxidation of broilers. Similarly, Farahat et al. [17] showed that flavonoid-rich plant extract can significantly increase liver GSH-Px content and reduce muscle MDA content, increasing antioxidant potential for broiler chickens.

Anthocyanin-rich blueberry extracts could improve lipid metabolism in mice by decreasing the microbiota in the intestine with the abundance of *Desulfovibrio* and increasing *Akkermansia*, *Bifidobacterium*, and *Lactobacillus* [37]. Hence, in the present study, the inclusion of anthocyanin-rich PCP could improve plasma lipid metabolism parameters, perhaps because anthocyanin could also regulate intestinal microbial flora in growing chickens. Of interest, HDL-C is “good cholesterol”, which can reduce the risk of heart attack by removing fatty deposits that can block the flow of blood in the arteries of the heart [38]. The results of this experiment suggest that PCP could increase HDL-C levels in the blood of Chishui black-bone chicken. The reason for this mechanism may be that anthocyanins could affect lipid hydrolysis, micelle formation, and cholesterol transfer to enterocytes of the initial process; in addition, anthocyanins could modulate the low-density lipoprotein receptor pathway, thereby increasing bile acid secretion and reducing cholesterol absorption [39]. These studies are consistent with Nasoetion et al. [40], who demonstrated that broilers receiving anthocyanin-rich purple sweet potato extract could have increased HDL-C concentrations in the blood and liver.

Chishui black-bone chicken is a unique local chicken breed in Guizhou with black (or dark) skin, meat, and membrane on bone. Thus, the meat color value is a grey area due to the black muscle. The difference in the number of melanocytes at different ages may have effects on the skin and meat color for the black-bone chicken [41]. The meat color did not differ for all groups, which may suggest that anthocyanins did not regulate related melanocyte gene expression in chickens but need more investigation to prove in further studies. It was of great value to observe influential factors on water holding capacity of meat for improving meat quality caused by reducing drip loss water loss rate in muscles [42]. Lipid oxidation in muscle can increase cell membrane permeability, thus decreasing hydrolysis sensitivity and reducing water reservation among myofibrils [43]. Flavonoid-rich plant extracts can affect meat quality by regulating the density, diameter, and cross-sectional area of muscle fibers [44]. Furthermore, anthocyanin plant extract can improve muscle antioxidant activity and reduce muscle free radicals to protect again lipid oxidation in animals [45]. Zdanowska-Ssiadek et al. [46] showed that the feeding of anthocyanin-rich herbal and vegetable additives could increase the breast muscle water holding capacity of chickens compared to the control group. Hence, the results of this study showed that dietary supplementation with anthocyanin-rich PCP reduced drip loss and water loss rate values in breast muscle, suggesting that anthocyanins can improve muscle water retention and meat quality characteristics, which is very beneficial to maintain the juiciness and taste of meat products from chickens. This may be due to the antioxidant properties of anthocyanins inhibiting the overexpression of forkhead transcription factors in muscle cells, thereby promoting the expression of muscle oxidative fibers and inhibiting lysogenic expression [45]. Our results are consistent with Pastsart and Pimpa [47], who reported that the addition of 0.8% anthocyanin-rich mangosteen (*Garcinia mangostana*) peel extract to the Ross 308 one-day-old female broiler chickens can reduce breast muscle drip loss and total water loss, improving the meat quality.

The type and content of amino acids directly affect the nutritional value and flavor of meat and are the main indicators for evaluating meat quality [48]. Some taste substances, including free amino acids (for example Glu), showed relatively high contents, thus resulting in them contributing to sensory perceived umami potential [49]. Vaithiyanathan et al. [50] suggested that polyphenol-rich plants can reduce thiobarbituric acid content and total volatile basic nitrogen in chickens. Moreover, free radicals readily react with highly susceptible amino acid residues, and the formation of protein carbonyls can lead to muscle protein oxidative damage [51]. Remarkably, Ji et al. [52] found electron donors and indicated hydrogen-bond interactions between ligands and amino acid main chains or side chains in the two-dimensional structure of anthocyanin-rich bilberry extract. Indeed, anthocyanin-rich plants might have high levels of crude protein and high amino acid concentrations [53,54]. Of interest, bioactive substances could enhance the umami sensory property by different interaction modes with the umami receptor [55]. Thus, the results of this study showed that the addition of PCP to the diet could increase the UAAs in the breast muscle of Chishui black-bone chickens. The reason might be that anthocyanin-rich plants showed strong antioxidant capacity and reduced free radical concentrations, thus improving amino acid levels in muscle [50]. Our results are in general agreement with the findings of Haščík et al. [56], who found that dietary supplementation with anthocyanin-rich probiotic and propolis extract could improve the amino acid concentrations of chicken meat. Additionally, Omar et al. [22] showed that phenolic-rich onion extracts can be used as a natural growth promoter in poultry feed because they can improve the growth rate of broiler chickens by improving the amino acid ileal digestibility of amino acids and intestinal histology.

It is well known that lipid oxidation status directly affects the quality of meat. The effect of fatty acids on shelf life depends on the proportion of unsaturated fatty acids (UFAs) [57]. Moreover, the higher the UFA concentrations are, the faster the fatty acid rate spoilage, and fatty acid products could accelerate the oxidation of myoglobin to methemoglobin, resulting in decreased meat quality in broiler chickens [58]. Some natural antioxidants can be used to delay the oxidation of fat and myoglobin in meat products [59]. Anthocyanins have strong antioxidant activity in vitro and can scavenge various reactive oxygen species, reactive nitrogen species, and chlorine species, as well as peroxynitrite and hypochlorous acid [60]. On the one hand, anthocyanins can terminate chain radical reactions, thereby preventing lipid oxidation in different lipid substrates [61]; on the other hand, anthocyanins can chelate metal ions and decompose peroxides by redox catalyzing metal ions [62]. Anthocyanins act as free radical scavengers and chain breakers, which can inhibit lipid peroxidation by providing donors of hydrogen atoms to PUFAs [63]. Thus, the results of this study found that feeding 80 mg/kg anthocyanin-rich PCP could improve muscle fatty acid concentrations, specifically PUFA levels, in growing Chishui black-bone chickens. These findings might be due to the special hydrogen in anthocyanins, which can quickly donate hydrogen atoms to lipid radicals and prevent lipid oxidation [64]. In addition, the fatty acid composition of chicken breast meat is dependent on genotype-related variation in fatty acid desaturase gene expression [65]. Jiang et al. [66] showed that the abundances of inflammation with gene expression of nuclear factor-kappa B and tumor necrosis factor-α were decreased, which could improve the lipid profile with fatty acid synthesis. Anthocyanins can upregulate the expression of antioxidant genes and downregulate the abundance of inflammatory gene expression in muscle [12]. This might also be an important reason why anthocyanins improve PUFA profiles by regulating related muscle antioxidant and inflammatory gene expression in chickens. Of interest, total PUFAs in the MA and HA groups were lower than those in the LA group, which may be due to the excessive addition of natural antioxidants that lead to the generation of peroxidation [67]. Consistent with our observations, Frasao et al. [68] demonstrated that dietary supplementation with anthocyanin-rich plant extract could improve muscle PUFA concentrations and could stabilize muscle oxidation in broiler meat. Similarly, Amer et al. [13] found that dietary addition of 280 mg/kg anthocyanin-rich roselle (*Hibiscus sabdariffa* L.) extract had the ability to enhance n-3 PUFA levels in the breast muscle of broiler chickens.

## 5. Conclusions

The current study indicated that dietary supplementation with anthocyanin-rich PCP not only increased growth performance and antioxidant capacity but also improved meat quality and muscle amino acid and fatty acid profiles, because (1) it can promote ADFI, BWC, and ADG; (2) it can enhance plasma TAC, SOD, GSH-Px, CAT, and Alb concentrations; (3) it could decrease muscle drip loss and water loss rate values; (4) it can increase muscle total EAA and UAA levels; and (5) it has the ability to improve fatty acid profiles in growing Chishui black-bone chickens. The optimum inclusion level of anthocyanin-rich PCP was 80 mg/kg under these experimental conditions.

## Figures and Tables

**Table 1:** Composition and nutrient levels of basal diets.

Ingredients	Chemical Composition, % of DM
Corn	61.50
Soybean meal	26.19
Soybean oil	1.05
Limestone	7.86
Fishmeal	0.10
Salt	0.30
Premix ^1^	3.00
Total	100
Nutrient levels	
Dry matter, % of the as-fed diet	93.35
Metabolizable energy, MJ/kg ^2^	12.12
Crude protein	16.02
Calcium	3.25
Total phosphorus	0.45
Available phosphorus	0.19
Lysine	0.87
Methionine	0.42
Methionine + cystine	0.72

^1^ Premix is provided per kg of diet: Cu 340 mg, Fe 2000 mg, Mn 2700 mg, Zn 2700 mg, Se 12 mg, vitamin E 850 IU, vitamin A 330,000 IU, vitamin D_3_ 133,500 IU, vitamin K_3_ 85 mg, nicotinamide 1200 mg, pantothenic acid 350 mg, vitamin K_3_ 85 mg, vitamin B_1_ 70 mg, vitamin B_2_ 200 mg, vitamin B_6_ 135 mg, vitamin B_12_ 0.8 mg, biotin 9 mg, choline chloride 12,000 mg, moisture 10%, methionine 5.0%. ^2^ Nutrient levels were measured values, whereas metabolizable energy was calculated value.

**Table 2:** Effect of anthocyanin-rich PCP on growth performance of growing chickens.

Items	PCP Supplemental Levels, mg/kg				SEM
	0	80	160	240	
ADFI, g/d	67.35 ^b^	73.05 ^a^	72.29 ^a^	71.45 ^a,b^	1.4959
Initial weight, g	919.36	940.11	946.48	934.49	13.4118
Final weight, g	1654.56 ^c^	1764.79 ^a^	1757.29 ^a,b^	1713.01 ^b^	17.0474
Body weight change, g	735.20 ^b^	824.68 ^a^	810.80 ^a^	778.51 ^a,b^	20.6924
ADG, g/d	9.80 ^b^	11.00 ^a^	10.81 ^a^	10.38 ^a,b^	0.2759
F/G	7.24	6.77	6.78	7.02	0.1869

Different letters within a row denote significant differences (*p* < 0.05). ADFI, average daily feed intake; ADG, average daily gain; F/G, feed/gain.

**Table 3:** Effect of anthocyanin-rich PCP on blood biochemical indexes of growing chickens.

Items	PCP Supplemental Levels, mg/kg				SEM
	0	80	160	240	
Antioxidant parameters					
TAC, U/mL	6.01 b	9.46 a	8.51 a	8.50 a	0.6312
SOD, U/mL	17.41 c	19.49 a	18.59 a,b	17.76 b,c	0.3752
GSH-Px, U/mL	170.61 c	217.57 a	214.99 a	194.70 b	6.6481
CAT, U/mL	7.24 b	9.27 a	7.79 a,b	7.27 b	0.5697
MDA, nmol/mL	9.67	6.74	8.36	8.68	1.0956
Lipid metabolism parameters					
T-CHO, mmol/L	2.40	1.44	1.61	1.98	0.4195
TG, mmol/L	0.73	1.29	1.65	1.10	0.8672
HDL-C, mmol/L	2.29 b	2.98 a	2.35 b	2.43 b	0.1340
LDL-C, mmol/L	1.28	2.04	1.25	1.60	0.4827
Immune parameters					
TP, μg/L	44.66	47.69	46.14	45.96	2.1965
Alb, g/L	14.79 ^b^	17.25 ^a^	18.55 ^a^	18.58 ^a^	0.7389

Different letters within a row denote significant differences (*p* < 0.05). TAC, total antioxidant capacity; SOD, superoxide dismutase; GSH-Px, glutathione peroxidase; CAT, catalase; MDA, malondialdehyde; T-CHO, total cholesterol; TG, triglyceride; LDL-C, low-density lipoprotein cholesterol; HDL-C, high-density lipoprotein cholesterol; TP, total protein; Alb, albumin.

**Table 4:** Effect of anthocyanin-rich PCP on meat quality of growing chickens.

Items	PCP Supplemental Levels, mg/kg				SEM
	0	80	160	240	
pH 45 min	5.65	4.98	5.88	4.93	0.9068
pH 24 h	4.94	4.87	5.73	4.90	0.8502
Meat color 45 min, L*	89.63	89.66	89.00	89.72	0.5767
Meat color 24 h, L*	89.51	89.18	90.00	90.00	0.4775
Drip loss, %	12.47 ^a^	11.13 ^b^	10.47 ^b^	10.94 ^b^	0.6356
Water loss rate, %	13.80 ^a^	10.03 ^a,b^	8.22 ^b^	8.06 ^b^	1.3755
Shear force, N	15.99	17.86	12.70	11.86	2.5865

Different letters within a row denote significant differences (*p* < 0.05). L*, L-value.

**Table 5:** Effect of anthocyanin-rich PCP on the amino acid of growing chickens.

Items, %	PCP Supplemental Levels, mg/kg				SEM
	0	80	160	240	
Asp	7.41 ^c^	7.87 ^b^	7.90 ^b^	8.03 ^a^	0.0317
Thr	3.53 ^c^	3.73 ^b^	3.77 ^b^	3.86 ^a^	0.0144
Ser	2.99 ^b^	3.23 ^a^	3.19 ^a^	3.24 ^a^	0.0136
Glu	11.65 ^b^	12.67 ^a^	12.62 ^a^	12.69 ^a^	0.0517
Gly	3.40 ^c^	3.45 ^b^	3.49 ^b^	3.68 ^a^	0.0144
Ala	4.46 ^c^	4.71 ^b^	4.72 ^b^	4.82 ^a^	0.0210
Val	3.93 ^c^	4.13 ^b^	4.14 ^b^	4.25 ^a^	0.0162
Met	2.03 ^c^	2.10 ^b^	2.20 ^a^	1.97 ^d^	0.0092
Ile	3.59 ^c^	3.87 ^a,b^	3.85 ^b^	3.93 ^a^	0.0209
Leu	6.13 ^c^	6.50 ^b^	6.53 ^b^	6.62 ^a^	0.0251
Tyr	2.70 ^c^	2.88 ^b^	2.90 ^a,b^	2.94 ^a^	0.0130
Phe	3.96 ^c^	4.14 ^b^	4.20 ^a^	4.25 ^a^	0.0180
His	3.36 ^c^	3.36 ^c^	3.65 ^b^	3.78 ^a^	0.0162
Lys	6.87 ^b^	7.36 ^a^	7.37 ^a^	7.46 ^a^	0.0315
Arg	4.94 ^c^	5.23 ^b^	5.24 ^b^	5.36 ^a^	0.0245
Pro	2.71 ^c^	2.83 ^b^	2.85 ^b^	2.96 ^a^	0.0161
EAA	30.02 ^c^	31.84 ^b^	32.05 ^a,b^	32.31 ^a^	0.1309
UAA	33.58 ^c^	35.72 ^b^	35.84 ^b^	36.41 ^a^	0.1482

Different letters within a row denote significant differences (*p* < 0.05). Asp, asparagine; Thr, threonine; Ser, serine; Glu, glutamic acid; Gly, glycine; Ala, alanine; Val, valine; Met, methionine; Ile, isoleucine; Leu, leucine; Tyr, tyrosine; Phe, phenylalanine; His, histidine; Lys, lysine; Arg, argnine; Pro, proline; EAA, essential amino acids; UAA, umami amino acids; SEM, standard error of mean. EAA = Thr + Val + Met + Ile + Leu + Phe + Lys; UAA = Asp + Glu + Gly + Ala + Tyr + Phe.

**Table 6:** Effect of anthocyanin-rich PCP on fatty acid of growing chickens.

Items, %	PCP Supplemental Levels, mg/kg				SEM
	0	80	160	240	
C12:0	0.054 ^a^	0.027 ^b^	0.029 ^b^	0.041 ^a,b^	0.0041
C14:0	1.005 ^a^	0.674 ^d^	0.755 ^c^	0.875 ^b^	0.0019
C14:1	0.228 ^a^	0.148 ^c^	0.180 ^b^	0.232 ^a^	0.0014
C15:0	0.097 ^a^	0.070 ^c^	0.066 ^d^	0.076 ^b^	0.0005
C16:0	27.799 ^b^	26.358 ^c^	28.977 ^a^	27.852 ^b^	0.0219
C16:1	5.595 ^a^	4.815 ^d^	5.440 ^b^	5.406 ^c^	0.0027
C17:0	0.215 ^a^	0.030 ^d^	0.054 ^c^	0.104 ^b^	0.0026
C18:0	7.759 ^b^	7.604 ^d^	8.065 ^a^	7.716 ^c^	0.0030
C18:1n9t	0.169 ^c^	0.182 ^b^	0.193 ^a^	0.186 ^b^	0.0013
C18:1n9c	35.733 ^d^	39.556 ^b^	39.850 ^a^	38.774 ^c^	0.0158
C18:2n6t	0.062	0.055	0.044	0.074	0.0115
C18:2n6c	17.768 ^b^	18.442 ^a^	14.349 ^d^	15.966 ^c^	0.0118
C20:0	0.063 ^b^	0.078 ^a^	0.065 ^b^	0.058 ^c^	0.0012
C18:3n6	0.114	0.061	0.062	0.218	0.0544
C20:1n9	0.178 ^a b^	0.279 ^a^	0.288 ^a^	0.089 ^b^	0.0503
C18:3n3	0.799 ^a^	0.684 ^c^	0.540 ^d^	0.711 ^b^	0.0032
C20:2	0.165 ^a^	0.145 ^b^	0.116 ^d^	0.129 ^c^	0.0012
C22:0	0.102 ^a^	0.052 ^d^	0.072 ^c^	0.085 ^b^	0.0010
C20:3n6	0.075 ^b^	0.028 ^d^	0.041 ^c^	0.186 ^a^	0.0024
Total SFAs	37.150 ^b^	34.894 ^d^	38.065 ^a^	36.806 ^c^	0.0235
Total MUFAs	41.868 ^c^	44.762 ^b^	45.725 ^a^	44.892 ^b^	0.1341
Total PUFAs	19.103 ^b^	19.633 ^a^	15.378 ^d^	17.358 ^c^	0.0126

Different letters within a row denote significant differences (*p* < 0.05). Total SFAs, sum of all the saturated fatty acid; total MUFAs, sum of all the monounsaturated fatty acid; total PUFAs, sum of all the polyunsaturated fatty acid.

## Data Availability

All data presented in the present research are available on request from the corresponding author.

## References

[1] del Bosque C.I.E., Grahl S., Nolte T., Mörlein D. (2022). Meat quality parameters, sensory properties and consumer acceptance of chicken meat from dual-purpose crossbreeds fed with regional faba beans. Foods.

[2] North M.K., Zotte A.D., Hoffman L.C. (2019). The use of dietary flavonoids in meat production: A review. Anim. Feed Sci. Technol..

[3] Rakesh S.U., Patil P.R., Mane S.R. (2010). Use of natural antioxidants to scavenge free radicals: A major cause of diseases. Int. J. PharmTech Res..

[4] Diaz-Sanchez S., D’Souza D., Biswas D., Hanning I. (2015). Botanical alternatives to antibiotics for use in organic poultry production. Poult. Sci..

[5] Umeno A., Horie M., Murotomi K., Nakajima Y., Youshida Y. (2016). Antioxdative and antidiabetic effects of natural polyphenols and isoflavones. Molecules.

[6] Lee N., Kwak H.S., Joo J.Y., Kang J., Lee Y. (2018). Effects of partial replacement of pork meat with chicken or duck meat on the texture, flavor, and consumer acceptance of sausage. J. Food Qual..

[7] Hashemipour H., Kermanshahi H., Golian A., Veldkamp T. (2013). Effect of thymol and carvacrol feed supplementation on performance, antioxidant enzyme activities, fatty acid composition, digestive enzyme activities, and immune response in broiler chickens. Poult. Sci..

[8] Roberto M., Antonio F., Luciana M., Paula S. (2020). Anthocyanins: A comprehensive review of their chemical properties and health effects on cardiovascular and neurodegenerative diseases. Molecules.

[9] Kšonžeková P., Mariychuk R., Eliašová A., Mudroñová D., Csank T., Király J., Marcinčáková D., Pistl J., Tkáčiková L. (2016). In vitro study of biological activities of anthocyanin-rich berry extracts on porcine intestinal epithelial cells. J. Sci. Food Agric..

[10] Tian X.Z., Paengkoum P., Paengkoum S., Chumpawadee S., Ban C., Thongpea S. (2019). *Short communication*: Purple corn (*Zea mays* L.) stover silage with abundant anthocyanins transferring anthocyanin composition to the milk and increasing antioxidant status of lactating dairy goats. J. Dairy Sci..

[11] Tian X.Z., Li J.X., Luo Q.Y., Zhou D., Long Q.M., Wang X., Lu Q., Wen G.L. (2021). Effects of purple corn anthocyanin on blood biochemical indexes, ruminal fluid fermentation, and rumen microbiota in goats. Front. Vet. Sci..

[12] Tian X.Z., Xin H.L., Paengkoum P., Siwaporn P., Ban C., Sorasak T. (2019). Effects of anthocyanin-rich purple corn (*Zea mays* L.) stover silage on nutrient utilization, rumen fermentation, plasma antioxidant capacity, and mammary gland gene expression in dairy goats. J. Anim. Sci..

[13] Amer S.A., Al-Khalaifah H.S., Gouda A., Osman A., Goda N.I.A., Mohammed H.A., Darwish M.I.M., Hassan A.M., Mohamed S.K.A. (2022). Potential effects of anthocyanin-rich roselle (*Hibiscus sabdariffa* L.) extract on the growth, intestinal histomorphology, blood biochemical parameters, and the immune status of broiler chickens. Antioxidants.

[14] Hajrawati H., Malaka R., Fatma F., Hakim M.R., Novita N., Suharyanto S. (2022). Evaluation of physicochemical properties and antioxidant activity of chicken meatballs by substitution of tapioca flour with purple sweet potato. Adv. Biol. Sci. Res..

[15] Tian X.Z., Lu Q., Paengkoum P., Paengkoum S. (2020). Short communication: Effect of purple corn pigment on change of anthocyanin composition and unsaturated fatty acids during milk storage. J. Dairy Sci..

[16] Jian H., Zu P., Rao Y., Li W., Mou T., Lin J., Zhang F. (2021). Comparative analysis of melanin deposition between chishui silky fowl and taihe silky fowl. J. Appl. Anim. Res..

[17] Farahat M.H., Abdallah F.M., Ali H.A., Hernandez-Santana A. (2016). Effect of dietary supplementation of grape seed extract on the growth performance, lipid profile, antioxidant status and immune response of broiler chickens. Animal.

[18] Tian X., Li J., Luo Q., Wang X., Wang T., Zhou D., Xie L., Ban C., Lu Q. (2022). Effects of purple corn anthocyanin on growth performance, meat quality, muscle antioxidant status, and fatty acid profiles in goats. Foods.

[19] (2004). Feeding Standard of Chicken.

[20] AOAC (1990). Official Methods of Analysis.

[21] (2016). National Food Safety Standard-Determination of Amino Acids in Food.

[22] Omar A.E., Al-Khalaifah H.S., Mohamed W.A.M., Gharib H.S.A., Osman A., Al-Gabri N.A., Amer S.A. (2020). Effects of phenolic-rich onion (*Allium cepa* L.) extract on the growth performance, behavior, intestinal histology, amino acid digestibility, antioxidant activity, and the immune status of broiler chickens. Front. Vet. Sci..

[23] (2016). National Food Safety Standard-Determination of Fatty Acids in Food.

[24] Changxing L., Chenling M., Alagawany M., Jianhua L., Dongfang D., Gaichao W., Wenyin Z., Sted S.F., Arain M.A., Saeed M. (2018). Health benefits and potential applications of anthocyanins in poultry feed industry. World Poult. Sci. J..

[25] Wang Y., Ma X., Ye J., Zhang S., Jiang S. (2021). Effects of dietary supplementation with bilberry extract on growth performance, immune function, antioxidant capacity, and meat quality of yellow-feathered chickens. Animals.

[26] Chamorro S., Viveros A., Rebole A., Rica B.D., Brenes A. (2015). Influence of dietary enzyme addition on polyphenol utilization and meat lipid oxidation of chicks fed grape pomace. Food Res. Int..

[27] Schiavone A., Guo K., Tassone S., Gasco L., Hernandez E., Denti R., Zoccarato I. (2008). Effects of a natural extract of breastnut wood on digestibility, performance traits, and nitrogen balance of broiler chicks. Poult. Sci..

[28] Halliwell B., Gutteridge M.C. (2015). Free Radicals in Biology and Medicine.

[29] Sihvo H.K., Immonen K., Puolanne E. (2014). Myodegeneration with fibrosis and regeneration in the pectoralis major muscle of broilers. Vet. Pathol..

[30] Starčević K., Krstulović L., Brozić D., Maurić M., Stojević Z., Mikulec Ž., Bajić M., Mašek T. (2015). Production performance, meat composition and oxidative susceptibility in broiler chicken fed with different phenolic compounds. J. Sci. Food Agric..

[31] Csernus B., Biró S., Babinszky L., Komlósi I., Jávor A., Stündl L., Remenyik J., Bai P., Oláh J., Pesti-Asbóth G. (2020). Effect of carotenoids, oligosaccharides and anthocyanins on growth performance, immunological parameters and intestinal morphology in broiler chickens challenged with *Escherichia coli* lipopolysaccharide. Animals.

[32] Tian X., Wang X., Li J., Luo Q., Ban C., Lu Q. (2022). The Effects of selenium on rumen fermentation parameters and microbial metagenome in goats. Fermentation.

[33] Small D.M., Coombes J.S., Bennett N., Johnson D.W., Gobe G.C. (2012). Oxidative stress, anti-oxidant therapies and chronic kidney disease. Nephrology.

[34] Lin L.Z., Sun J., Chen P., Harnly J. (2011). UHPLC-PDA-ESI/HRMS/MS^n^ analysis of anthocyanins, flavonol glycosides, and hydroxvcinnamic acid derivatives in red mustard greens (*Brassica juncea* Coss variety). J. Agric. Food Chem..

[35] Tian X.Z., Lu Q., Zhao S.G., Li J.X., Luo Q.Y., Wang X., Zhang Y.D., Zheng N. (2021). Purple corn anthocyanin affects lipid mechanism, flavor compound profiles, and related gene expression of *longissimus thoracis et lumborum* muscle in goats. Animals.

[36] Chamorro S., Viveros A., Centeno C., Romero C., Arija I., Brenes A. (2013). Effects of dietary grape seed extract on growth performance, amino acid digestibility and plasma lipids and mineral content in broiler chicks. Animal.

[37] Guo J., Han X., Tan H., Huang W., You Y., Zhan J. (2019). Blueberry extract improves obesity through regulation of the gut microbiota and bile acids via pathways involving FXR and TGR5. iScience.

[38] Young C.E., Karas R.H., Kuvin J.T. (2004). High-density lipoprotein cholesterol and coronary heart disease. Cardiol. Rev..

[39] Yao S.L., Xu Y., Zhang Y.Y., Lu Y.H. (2013). Black rice and anthocyanins induce inhibition of cholesterol absorption in vitro. Food Funct..

[40] Nasoetion M.H., Atmomarsono U., Sunarti D., Suthama N. (2019). Growth performance and lipid profile of broilers fed different levels of purple sweet potato extract and raised under different stocking densities. Livest. Res. Rural Dev..

[41] Khumpeerawat P., Doungjinda M., Phasuk Y. (2021). Factors affecting gene expression associated with the skin color of black-bone chicken in thailand. Poult. Sci..

[42] Mir N.A., Rafiq A., Kumar F., Singh V., Shukla V. (2017). Determinants of broiler chicken meat quality and factors affecting them: A review. J. Food Sci. Technol..

[43] Wang Z.G., Pan X.J., Peng Z.Q., Zhao R.Q., Zhou G.H. (2009). Methionine and selenium yeast supplementation of the maternal diets affects color, water-holding capacity, and oxidative stability of their male offspring meat at the early stage. Poult. Sci..

[44] Zhou Y., Ruan Z., Li X.L., Mi S.M., Yin Y.L. (2016). *Eucommia ulmoides* Oliver leaf polyphenol supplementation improves meat quality and regulates myofiber type in finishing pigs. J. Anim. Sci..

[45] Lee S.H., Joo S.T., Ryu Y.C. (2010). Skeletal muscle fiber type and myofibrillar proteins in relation to meat quality. Meat Sci..

[46] Zdanowska-Ssiadek A., Lipińska-Palka P., Damaziak K., Michalczuk M., Marchewka J. (2019). Antioxidant effects of phytogenic herbal-vegetable mixtures additives used in chicken feed on breast meat quality. Anim. Sci. Pap. Rep..

[47] Pastsart U., Pimpa O. (2021). Growth performance, meat quality, meat oxidation and intestinal bacterial contents of broilers fed with *Garcinia* mangostana peel extract. Indian J. Anim. Res..

[48] Pripis-Nicolau L., Revel G.D., Bertrand A., Maujean A. (2000). Formation of flavor components by the reaction of amino acid and carbonyl compounds in mild conditions. J. Agric. Food Chem..

[49] Mouritsen O.G., Duelund L., Petersen M.A., Mikael A., Hartmann A.L., Frøst M.B. (2019). Umami taste, free amino acid composition, and volatile compounds of brown seaweeds. J. Appl. Phycol..

[50] Vaithiyanathan S., Naveena B.M., Muthukumar M., Girish P.S., Kondaiah N. (2011). Effect of dipping in pomegranate (*Punica granatum*) fruit juice phenolic solution on the shelf life of chicken meat under refrigerated storage (4 °C). Meat Sci..

[51] Estévez M. (2015). Oxidative damage to poultry: From farm to fork. Poult. Sci..

[52] Ji Y., Liu D., Jin Y., Zhao J., Wang H. (2021). *In vitro* and *in vivo* inhibitory effect of anthocyanin-rich bilberry extract on α-glucosidase and α-amylase. LWT Food Sci. Technol..

[53] Tian X.Z., Paengkoum P., Paengkoum S., Thongpe S., Ban C. (2018). Comparison of forage yield, silage fermentative quality, anthocyanin stability, antioxidant activity, and *in vitro* rumen fermentation of anthocyanin-rich purple corn (*Zea mays* L.) stover and sticky corn stover. J. Integr. Agric..

[54] Dancs G., Kondrák M., Bánfalvi Z. (2008). The effects of enhanced methionine synthesis on amino acid and anthocyanin content of potato tubers. BMC Plant Biol..

[55] Wang W., Zhou X., Liu Y. (2020). Characterization and evaluation of umami taste: A review. TrAC Trends Anal. Chem..

[56] Haščík P., Pavelková A., Tkáová J., Čuboň J., Kačániová M., Habánová M., Mlyneková E. (2020). The amino acid profile of broiler chicken meat after dietary administration of bee products and probiotics. Biologia.

[57] Turcu R.P., Panaite T.D., Untea A.E., Vlaicu P.A., Mironeasa S. (2021). Effects of grape seed oil supplementation to broilers diets on growth performance, meat fatty acids, health lipid indices and lipid oxidation parameters. Agriculture.

[58] Ma J., Wang J., Mahfuz S., Long S., Wu D., Gao J., Piao X. (2021). Supplementation of mixed organic acids improves growth performance, meat quality, gut morphology and volatile fatty acids of broiler chicken. Animals.

[59] Ribeiro J.S., Jose M., Silva L.R., Pereira L.L., Santo I.A., Lannes S.C.S., Silva M.V.D. (2019). Natural antioxidants used in meat products: A brief review. Meat Sci..

[60] Hernández I., Alegre L., Breusegem F.V., Munné-Bosch S. (2009). How relevant are flavonoids as antioxidants in plants. Trends Plant Sci..

[61] Maqsood S., Benjakul S., Abushelaibi A., Alam A. (2014). Phenolic compounds and plant phenolic extracts as natural antioxidants in prevention of lipid oxidation in seafood: A detailed review. Compr. Rev. Food Sci. Food Saf..

[62] Maqsood S., Benjakul S. (2010). Comparative studies of four different phenolic compounds on *in vitro* antioxidative activity and the preventive effect on lipid oxidation of fish oil emulsion and fish mince. Food Chem..

[63] Seeram N.P., Nair M.G. (2002). Inhibition of lipid peroxidation and structure−activity-related studies of the dietary constituents anthocyanins, anthocyanidins, and catechins. J. Agric. Food Chem..

[64] Tian X.Z., Wang X., Ban C., Luo Q.Y., Li J.X., Lu Q. (2022). Effect of purple corn anthocyanin on antioxidant activity, volatile compound and sensory property in milk during storage and light prevention. Front. Nutr..

[65] Boschetn E., Bordoni A., Meluzzi A., Castellini C., Bosco A.D., Sirri F. (2016). Fatty acid composition of chicken breast meat is dependent on genotype-related variation of FADS, and FADS2 gene expression and desaturating activity. Animal.

[66] Jiang H., Zhang W., Li X., Xu Y., Jiang W. (2021). The anti-obesogenic effects of dietary berry fruits: A review. Food Res. Int..

[67] Tian X.Z., Li J.X., Luo Q.Y., Wang X., Xiao M.M., Zhou D., Lu Q., Chen X. (2022). Effect of supplementation with selenium-yeast on muscle antioxidant activity, meat quality, fatty acids and amino acids in goats. Front. Vet. Sci..

[68] Frasao B., Rosario A., Rodrigues B.L., Bitti H.A., Conte-Junior C.A. (2021). Impact of juara (*Euterpe edulis*) fruit waste extracts on the quality of conventional and antibiotic-free broiler meat. Poult. Sci..

